# Soil properties and productivity in two long-term crop rotations differing with respect to organic matter management on an Albic Luvisol

**DOI:** 10.1038/s41598-018-37087-4

**Published:** 2019-02-12

**Authors:** S. Martyniuk, D. Pikuła, M. Kozieł

**Affiliations:** 1 Department of Agricultural Microbiology, Institute of Soil Science and Plant Cultivation, Czartoryskich 8, 24-100 Puławy, Poland; 2 Department of Plant Nutrition and Fertilization, Institute of Soil Science and Plant Cultivation, Czartoryskich 8, 24-100 Puławy, Poland

## Abstract

This study was based on a three-factor experiment carried out since 1980 on a loamy sand (Albic Luvisol) in which arable crops were grown in two 4-years rotations: RotA (grain maize, winter wheat, spring barley and silage maize) and RotB [grain maize, winter wheat plus mustard green manure (GM), spring barley and grass–clover ley (GCL)]. The soil in RotB with an increased input of OM (GM and 1-year GCL) accumulated significantly larger amounts of soil organic carbon and soil microbial biomass C, had higher activities of dehydrogenase and acid phosphatase enzymes and gave significantly higher winter wheat grain yields compared to the soil in RotA. However, in the absence of liming, the soil in RotB, contrary to that in RotA, became more acidic, had reduced activity of alkaline phosphatase and lower contents of Ca and Mg, and contained a diminished proportion of the >0.5 mm macroaggregates fraction. These soil deteriorative effects of crop rotations delivering larger amounts of OM have not been reported so far. In both rotations FYM applied once per 4-year rotation at 40 Mg ha^−1^ improved all the tested soil properties and had mitigating effects on the negative changes found in the soil of RotB.

## Introduction

Biological, chemical and physical properties of mineral soils depend to a great extent on the content of soil organic carbon (SOC) in their matrix and for this reason SOC, the main component of soil organic matter (SOM), is regarded as an integrative and most important indicator of soil quality^1–3^. The quantity and quality of SOM in soils depend on many edaphic and environmental factors, and change in response to various agriculture related practices such as: soil tillage, organic amendments, crop rotation and mineral fertilization. Crop management practices that help to maintain or enrich cultivated soils in organic matter (OM) include those which restrict mineralization of SOC, e.g. reduced tillage intensity^4,5^, and those which increase inputs and gains of OM in soils, such as: applications of organic fertilizers and wastes (animal manures, green manures, composts, sewage sludge)^2,3,6–9^, diversified crop rotations^2–4^, particularly those including grass-legume leys, and balanced mineral fertilization increasing crop residues^4,7^. Interactive effects of organic amendments, crop rotations and mineral fertilizers on SOM accumulation and other soil properties have been extensively studied, also in numerous long-term field experiments^2,4,10–13^, however green manures were seldom included in these experiments. Green manuring (GM) is most often practiced by ploughing down biomass of cover/catch crops in order to enrich soils in OM, to improve soil structure and productivity^4,8,14^, to stimulate activity of soil dwelling organisms^9,15^, and to reduce soil erosion, including nutrients leaching^8,14^. Thus, all good crop and soil management practices leading to the accumulation OM in soils can be regarded as beneficial to their quality^2,7–12^. However, it has also been shown that large inputs of OM, e.g. through long-term applications of high rates of cattle manure or manure + NPK, resulted in the deterioration of the formation and stability of large soil aggregates^13,16^.

With respect to other soil properties, it was shown that externally added plant materials, like green manures or composts, elevated soil pH^2,17^. However, we have found in our previous studies^18^ that after 16 years of growing arable plants in a cropping system with an increased input of OM, due to growing grass-clover ley (GCL) and mustard green manure (GM), soil pH decreased in comparison to that in similar cropping system, but without GCL and GM. This long-term field experiment is still running and the main objectives of the present study were (i) to characterize changes in biological, chemical and physical soil properties after 33 years of growing arable crops in two 4-years rotations differing primarily with respect to organic matter management systems and (ii) to elucidate possible drivers of these changes.

## Results and Discussion

### Organic matter input

The long-term field experiment (initiated in 1980) which provided the results presented in this work includes two 4-years crop rotations (RotA and RotB) with the following order of crops: grain maize, winter wheat, spring barley and silage maize – for RotA and: grain maize, winter wheat plus mustard GM, spring barley (with undersown grass–clover) and grass–clover ley (GCL) – for RotB. These rotation fields have been divided into four replicated blocks and within each block different application rates of FYM and inorganic N fertilized were varied in a split-plot design. All soil amendments applied in this experiment, particularly mineral N fertilizer and FYM rates, were the same in both rotations (Table 1). However, the compared crop rotations differed substantially in organic matter management systems, particularly with respect to fresh organic matter input, which was markedly larger in RotB than in RotA as a result of growing mustard GM (after winter wheat) and GCL in the former one. With the exception of the year 2013, when mustard yields were low due to the dry autumn in this year, in previous years mustard above-ground biomass ranged from about 3 Mg ha^−1^ fresh weight (FW) in the unfertilized treatments to more than 20 Mg of FW ha^−1^ (3.1 DM ha^−1^) in the treatments with the highest rates of FYM and mineral N fertilizer (Table 2). We did not measure biomass yields of mustard roots, but results of other studies^19^ indicate that at approximately 20 Mg FW ha^−1^ of mustard above-ground parts, this plant provides also about 2.5 Mg FW ha^−1^ of roots. Thus, depending on the fertilization treatments, the soil in RotB receives, per rotation, from about 6 Mg ha^−1^ to 23 Mg ha^−1^ of fresh organic matter (mustard) more than that in RotA. As the forth crop in each rotation cycle, silage maize or GCL are grown in RotA and RotB, respectively. It was estimated that incorporation of grass leys or GCLs can provide soils with up to 10 Mg ha^−1^ of dry organic matter^20,21^. On the other hand, comparisons made by Jarchow & Liebman^21^ indicate that corn can produce approximately four to six times less root biomass than C3 grasses or grass-legume mixtures. Taking these results into considerations, it seems reasonable to assume that GCLs enrich the soil of RotB in considerably larger amounts of fresh organic matter (residues of roots and lower parts of stems) than silage maize grown in RotA. This increased and long-lasting input of organic matter (GM and GCL) in RotB has caused both beneficial and also some negative changes in selected soil properties and winter wheat yields obtained in this rotation as compared to those in RotA.

**Table 1:** Inorganic fertilizer rates applied in RotA and RotB of the long-term experiment at Grabow, Poland.

Crop rotation	Plant	Inorganic fertilizer rate (kg ha^−1^ y^−1^)					
		N0	N1^b^	N2	N3	P_2_O_5_	K_2_O
RotA	Grain maize	0	50	100	150	54	160
	Winter wheat	0	50	100	150	54	100
	Spring barley	0	30	60	90	54	85
	Maize for silage	0	50	100	150	54	120
RotB	Grain maize^a^	0	50	100	150	54	160
	Winter wheat	0	50	100	150	54	100
	Spring barley	0	30	60	90	54	85
	Grass-clover ley	0	50	100	150	54	120

**Table 2:** Yields (Mg ha^−1)^ of mustard green manure fresh and dry matter (in brackets) grown after winter wheat harvest in Rot B of the long-term experiment at Grabow, Poland.

FYM (Mg ha^−1^)	N rates (kg ha^−1^)	1989	1993	1997	2001	2005	2009	2013
0	0	7.65 (1.45)	6.31 (0.88)	9.20 (1.79)	7.45 (1.69)	5.10 (1.22)	3.50 (1.18)	0.50 (0.10)
0	100	14.71 (2.48)	17.58 (2.36)	13.00 (2.28)	13.55 (2.81)	12.00 (2.45)	3.20 (0.88)	0.60 (0.20)
0	150	15.77 (2.42)	18.85 (2.40)	13.60 (2.36)	14.85 (2.50)	13.20 (2.30)	4.80 (1.09)	0.50 (0.10)
40	0	8.18 (1.53)	6.30 (0.85)	9.60 (1.70)	8.65 (2.05)	7.40 (1.83)	5.30 (1.65)	0.50 (0.20)
40	100	15.73 (2.64)	18.08 (2.38)	12.60 (2.34)	16.50 (3.21)	12.90 (2.63)	5.30 (1.55)	0.50 (0.20)
40	150	17.50 (2.66)	19.30 (2.52)	12.80 (2.35)	20.40 (3.10)	14.80 (2.87)	7.20 (2.02)	0.50 (0.20)
Mean		13.26 (2.20)	14.40 (1.90)	11.80 (2.14)	13.57 (2.56)	10.90 (2.23)	4.88 (1.44)	0.52 (0.17)

### Beneficial effects

**Figure 1:**
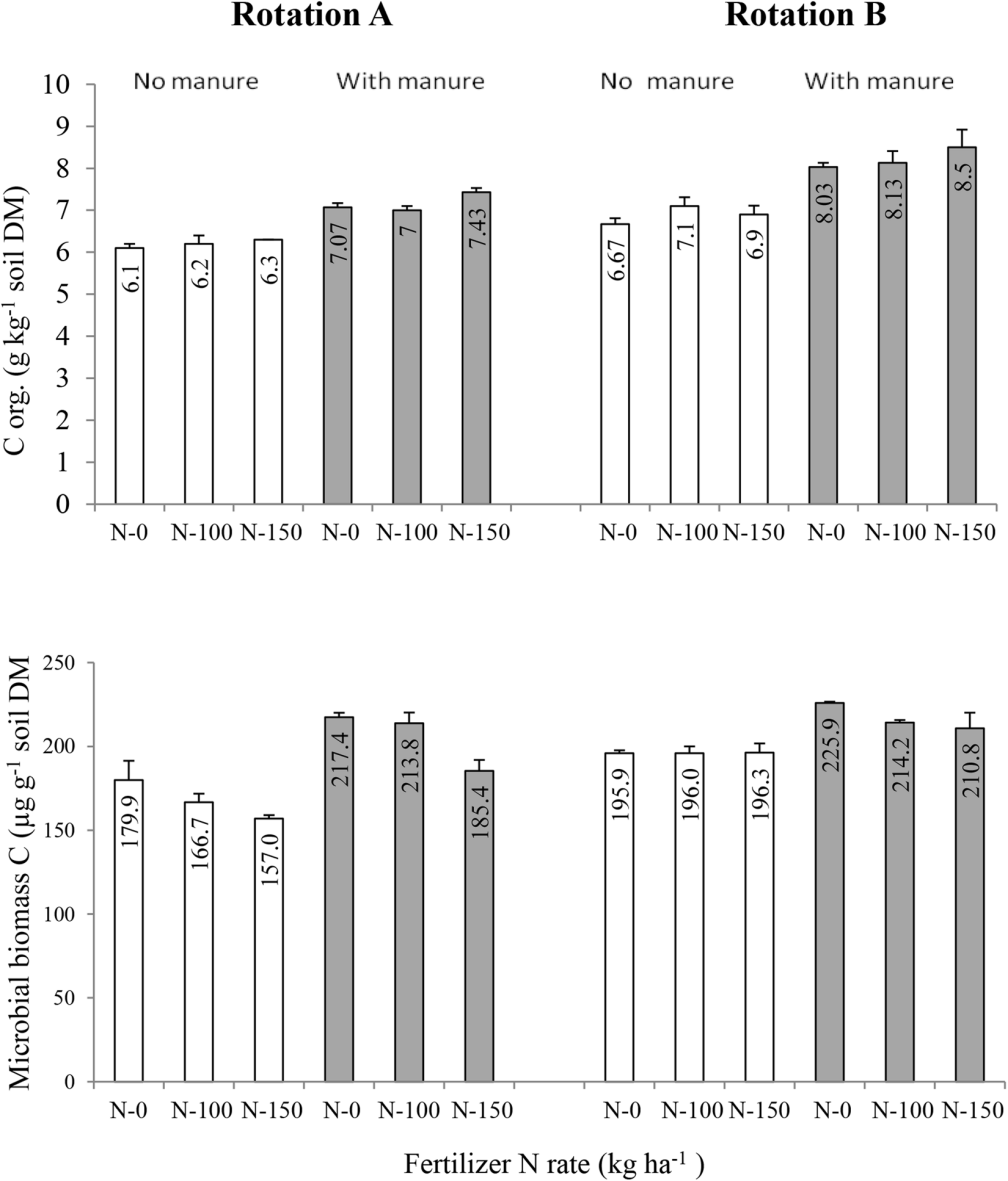
Organic C and microbial biomass C contents in soil as influenced by crop rotation (I), manure application (II) and mineral N rate (III) in the long-term field experiment at Grabow, Poland. Error bars denote standard deviations (n = 3). LSD(*P* ≤ 0.05): for C org.: I = 0.52, II = 0.65, III = 0.23, Interactions = not significant; for microbial biomass C: I = 11.5, II = 10.1, III = 11.2, Interactions = not significant.

The soil in RotB receiving higher amounts of OM accumulated significantly more SOC than the soil in RotA, irrespective of FYM application and mineral N rates (Fig. 1). Averaged across all the treatments, the SOC content in RotB was almost 12% higher than that in RotA. In RotA the unamended soil (N-0, no FYM) contained the lowest amount (6.1 g kg^−1^) of SOC, while the highest content of SOC (7.43 g kg^−1^) was found in the soil fertilized with N-150 and FYM. In RotB the corresponding values of SOC were 6.67 g kg^−1^ and 8.5 g kg^−1^, respectively. In both rotations and at all N rates soil amendment with 40 Mg ha^−1^ of FYM resulted in significantly higher SOC contents compared to the unmanured soil. The soil amended with 40 Mg ha^−1^ of FYM in RotA was 13.5% richer in SOC, on average for N rates, than the unmanured soil. In RotB this increase was significantly higher and amounted to 16.2%. In general, mineral N fertilization had also a beneficial effect on SOC accumulation. This effect was insignificant when FYM was not applied in RotA, but in RotB in the absence of FYM the soil treated with 100 and 150 kg N ha^−1^ contained significantly larger amounts of SOC than the unfertilized soil (N-0). When manure was used, application of 150 kg N ha^−1^ resulted in the accumulation of the maximum amounts of SOC in both rotation (Fig. 1).

Soil microbial biomass C (SMBC) contents and soil enzymes (dehydrogenase, acid phosphatase) activities were also significantly affected by all the treatments studied in this experiment (Fig. 1, Table 3). In general, these parameters had significantly higher values in RotB than in RotA and when soil was amended with FYM compared to the unmanured soil in both rotations. The exception was the acid phosphatase activity, which was significantly higher in the unmanured soil at N-0 than in the soil amended with FYM at N-0, but only in RotB (Table 3). SMBC was unaffected by mineral N rates only in the case of the unmanured soil in RotB, but in all other treatments microbial biomass C decreased as N rates increased (Fig. 1). Similar responses to N rates were also found for dehydrogenase activities, but in the case of this enzyme its activity was not significantly affected by N rates only in the unmanured soil of RotA (Table 3). Although the overall effect of N fertilization on the activity of acid phosphatase was insignificant, interactions were significant (Table 3). Irrespective of FYM application in RotA, both N rates were beneficial for the activity of this enzyme, particularly in comparison to the N-0 treatment, but in the case of RotB the opposite was true, especially in the unmanured soil. The results shown above are in accord with those reported earlier in numerous studies all over the world, which clearly indicate that various organic amendments (FYM, slurry, composts, straw and other plan residues) and diversified crop rotations, in particular those comprising grasses or grass-legume leys, beneficially affect different soil processes and properties, including accumulation of SOC, SMBC and stimulation of soil biological activities^2–8,11^. There is also a general consensus that proper soil fertility management practices on croplands, particularly with respect to N fertilization, enhance SOC sequestration and are beneficial for soil microorganisms and their activity, mainly due to a greater return of crop residues to the soil^3,4,7,22,23^. However, there are also reports showing that mineral N fertilizers, particularly their high rates, can reduce SMBC and soil enzymes activities^5,24^, and our results with respect to SMBC and the dehydrogenase activity (Fig. 1, Table 3) are in accordance with these reports.

**Table 3:** Activity of acid and alkaline phosphatase (µg *p*NP g^−1^ h^−1^) and dehydrogenase (µg formazan g^−1^ 24 h^−1^) in soil as influenced by crop rotation (I), manure application (II) and N fertilizer rates (III) in the long-term experiment at Grabow, Poland.

Crop rotation	Amendments		Enzymes		
	Manure (Mg ha^−1^)	N mineral (kg ha^−1^)	Alkaline phosphatase	Acid phosphatase	Dehydrogenase
A (grain maize – winter wheat – spring barley – silage maize)	0	0	8.63	33.23	17.28
	0	100	6.57	37.53	16.80
	0	150	5.57	38.06	16.38
	Mean		6.92	36.28	16.81
	40	0	14.63	47.87	35.67
	40	100	11.77	51.77	29.50
	40	150	9.70	55.67	27.07
	Mean		12.03	51.77	30.74
	Overall mean		9.48	44.02	23.78
B (grain maize – winter wheat^†^ – spring barley – GCL^‡^)	0	0	6.66	69.57	33.07
	0	100	6.03	59.03	27.07
	0	150	4.63	51.87	18.47
	Mean		5.77	60.17	26.20
	40	0	11.0	62.97	41.17
	40	100	8.43	62.50	31.43
	40	150	6.60	61.80	23.43
	Mean		8.68	62.42	32.01
	Overall mean		7.22	61.30	29.11
LSD (*P* ≤ 0.05)			For: I = 1.22, II = 0.58, III = 0.61 Interactions: II/I = 0.82 III/I = n.s. III/II = 0.86	For: I = 3.72, II = 2.48, III = n.s. Interactions: II/I = 3.29 III/I = 3.11 III/II = 3.11	For: I = 3.09, II = 1.47, III = 1.76. Interactions: II/I = 2.08 III/I = 2.49 III/II = 2.40

^†^plus mustard green manure; ^‡^grass-clover ley.

**Figure 2:**
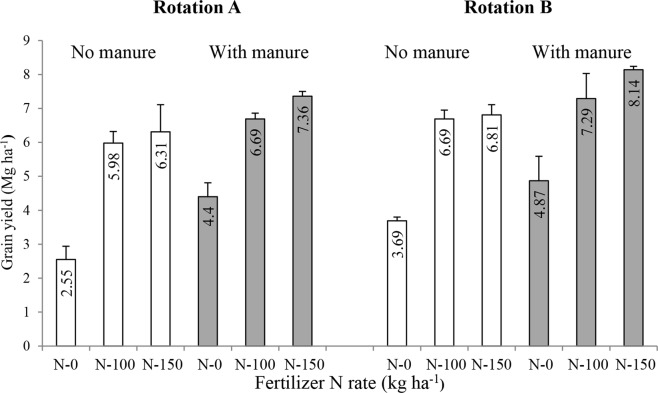
Winter wheat grain yield as influenced by crop rotation (I), manure application (II) and mineral N rate (III) in the long-term field experiment at Grabow, Poland. Error bars denote standard deviations (n = 4). LSD(*P* ≤ 0.05): for I = 0.34, II = 0.30, III = 0.29, Interactions: I x II and I x III = not significant, II x III = 0.40.

**Figure 3:**
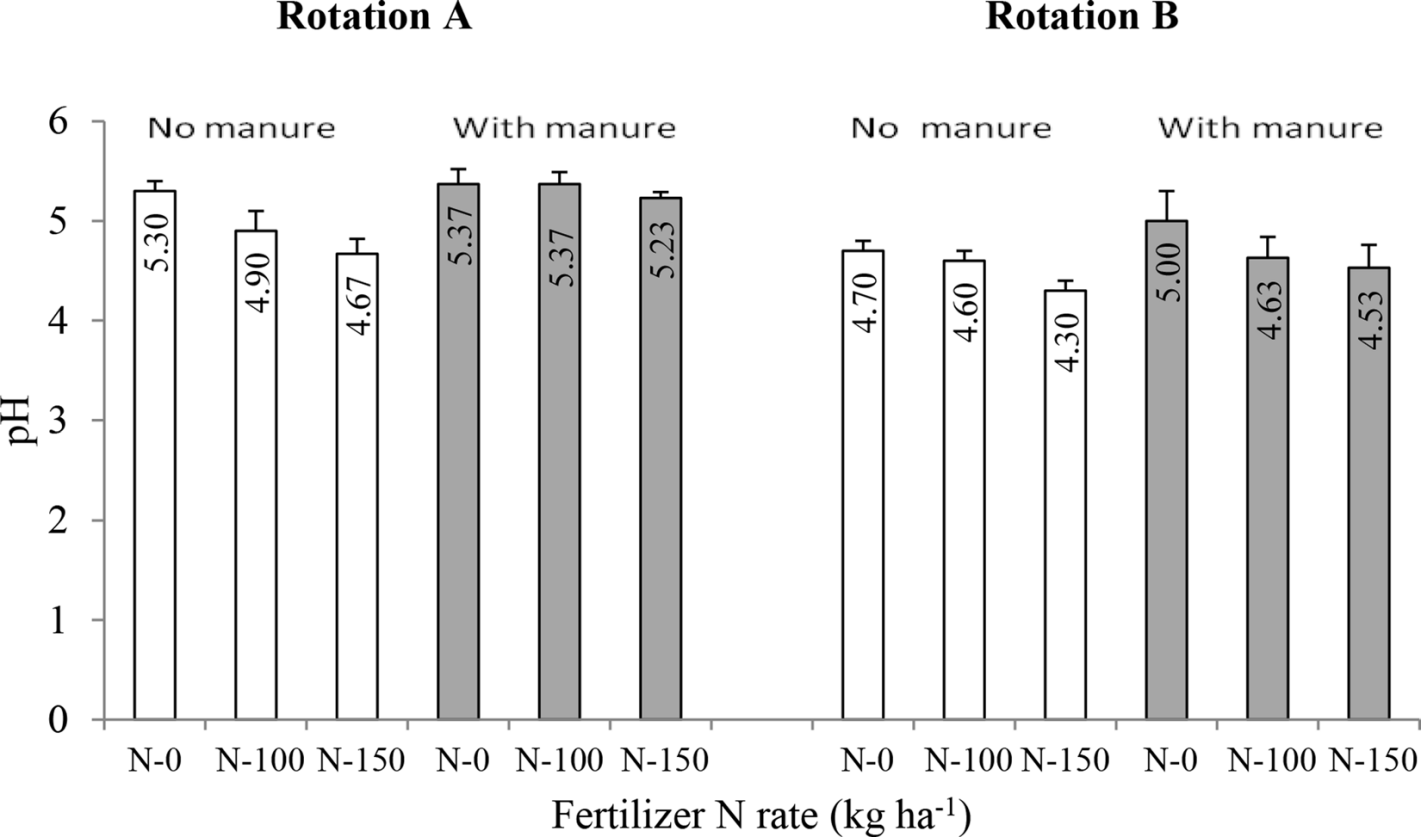
Soil acidity (pH in H_2_O) as influenced by crop rotation (I), manure application (II) and mineral N rate (III) in the long-term field experiment at Grabow, Poland. Error bars denote standard deviations (n = 3). LSD(*P* ≤ 0.05): for I = 0.31, II = 0.20, III = 0.09, Interactions: I x II and I x III = not significant, II x III = 0.13.

In 2013 winter wheat was grown in both rotations and it gave significantly higher grain yields in all the treatments of RotB than in the corresponding treatments of RotA (Fig. 2), even though mineral fertilization (NPK) was the same in these rotations (Table 1). Ten Berge *et al*.^25^ reported that mean DM yields of all other crops grown in this experiment were also higher in RotB than in RotA. The lowest grain yield (2.55 Mg ha^−1^) was obtained in RotA on plots without FYM and N application, while in the same treatment in RotB winter wheat yielded over 1 Mg ha^−1^ more. Grain yields on the manured plots at N-0 were 4.4 Mg ha^−1^ and 4.87 Mg ha^−1^ for RotA and RotB, respectively. Beneficial effects of FYM addition on winter wheat yields were also clearly visible at both N rates (N-100 and N-150). In both rotations the maximum grain yields were obtained at the highest N rate (150 kg ha^−1^) and FYM application, but in RotB the yield was almost 10% higher than that in RotA (Fig. 2). The higher SMBC content (Fig. 1) and more active microorganisms, as indicated by the increased dehydrogenase and acid phosphatase activities (Table 3), transforming larger amounts of fresh OM and the stored SOC (Fig. 1) in RotB were probably responsible for a better performance of winter wheat (Fig. 2) and other crops grown in this rotation than those grown in RotA^25^, despite less favorable soil pH in the former one (Fig. 3). The lack of yield reduction in response to soil acidification is uncommon, although not unknown in the scientific literature. For example, Schroder *et al*.^26^ monitored soil acidification as a result of over 30 years use of increasing rates (up to 272 kg N ha^−1^) of various N mineral fertilizers and found that although soil pH levels decreased to <5.0 already after 10 years of N fertilization, no significant reductions of winter wheat grain yield were observed during 25 consecutive growing seasons. It is important to add that in this experiment winter wheat was grown in monoculture and no organic amendments were added to the soil. It seems reasonable to assume that in our experiment yield decreases will occur in the future and that the delay in the appearance of negative effects of a stronger soil acidification in RotB on crop yields results from the beneficial effects (as discussed above) of a higher input of organic matter (fresh GM, GCL) in this rotation as compared to that in RotA.

### Negative effects

In this experiment no soil liming was performed to show soil buffering properties of FYM, and Fig. 3 shows that this effect was evident in both rotations, although less pronounced in RotB. For the purpose of this study only treatments with 40 Mg ha^−1^ of FYM were selected, as similar rates (30–40 Mg ha^−1^) are most often used in practice by farmers and also because higher rates (60 Mg ha^−1^ and 80 Mg ha^−1^) of FYM applied in this experiment were only slightly more effective in maintaining soil pH than FYM at 40 Mg ha^−1^. The soil pH values in the case of unmanured plots of RotA ranged from 5.3 in the N-0 treatment to 4.9 and 4.67 when 100 kg ha^−1^ and 150 kg ha^−1^ of N were applied, respectively. Soil pH in the unmanured plots of RotB had significantly lower values, which were 4.7, 4.6 and 4.3 for N-0, N-100 and N-150 treatments, respectively (Fig. 3). In the absence of liming increasing fertilizer N rates caused stronger soil acidification, particularly in the unmanured plots of both rotations, thus confirming results of previous studies^5,27-29^.

**Figure 4:**
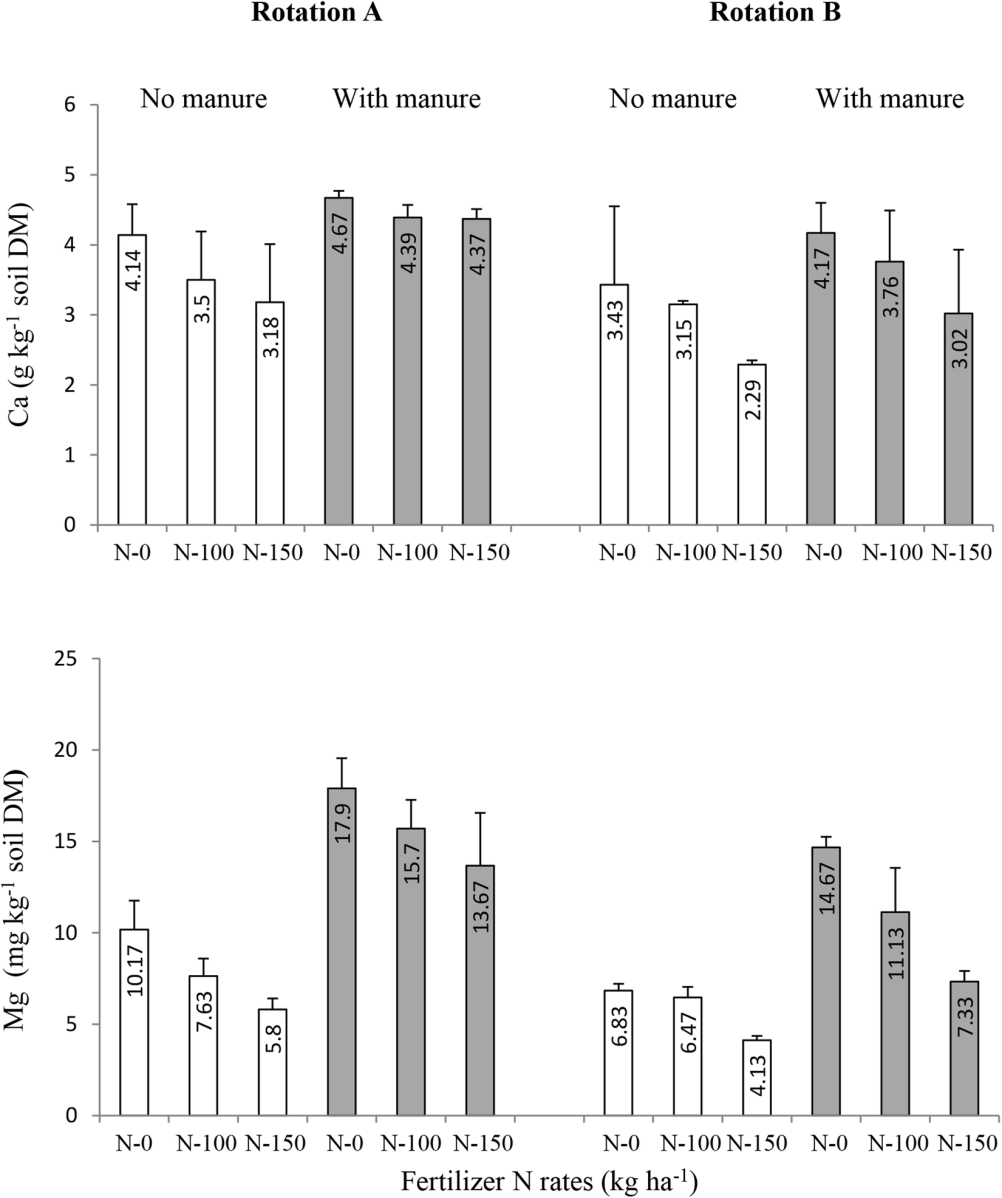
Ca and Mg contents in soil as influenced by crop rotation (I), manure application (II) and mineral N rate (III) in the long-term field experiment at Grabow, Poland. Error bars denote standard deviations (n = 3). LSD(*P* ≤ 0.05): for Ca: I = 0.32, II = 0.43, III = 0.36, Interactions = not significant; for Mg: I = 2.11, II = 2.02, III = 1.25, Interactions = not significant.

Since soil amendments, particularly mineral N fertilizer and FYM rates, were the same in both rotations we attribute the lower values of soil pH in RotB to growing mustard GM and GCL in this rotation, which had beneficial effects on crop yields, as discussed above. Higher crop yields^25^ (Fig. 2) indicate higher plant offtake and export of nutrients (including basic cations (K^+^, Ca^2+^, Mg^2+^), from the soil in RotB than that in RotA, and this was probably the main cause for the more intensive soil acidification in RotB compared to RotA (Fig. 3). Indeed, Fig. 4 shows that the exchangeable Ca and Mg contents in the soil of all the treatments in RotB were significantly lower than those in RotA. Although the chemical mechanisms for soil pH change by plant residues are not fully understood, it is generally accepted that the most significant proton (H^+^) and hydroxyl ion (OH^−^) generating processes occur during C and N transformations in soils^27,29^. CO_2_ production during microbial decomposition of fresh organic matter (GM and GCL) in Rot B might have been another soil acidifying factor in this rotation.

Contrary to the acid phosphatase, the activity of alkaline phosphatase was significantly lower in all the treatments of RotB compared to RotA (Table 3), probably due to more acidic soil reaction in RotB than that in RotA^30^. In both rotations soil manuring had beneficial effects on the alkaline phosphatase, but increasing rates of fertilizer N significantly reduced the activity of this enzyme and these results agree with those obtained in other studies^5,31,32^.

To get insight into the soil structure (aggregation) as influenced by the experimental factors we have sieved dry soil samples through a set of sieves to separate two macroaggregate fractions (>0.5 mm and 0.5–0.25 mm) and one microaggregate fraction (<0.25 mm) and results of these analyses are shown in Table 4. The soil in RotA contained significantly more of the >0.5 mm macroaggregate fraction than the soil in RotB, but the opposite was true for the smaller (0.5–0.25 mm) macroaggregates and the microaggregate fraction, indicating that the formation of large aggregates in RotB was reduced. Although it has been reported that in soils treated yearly and for a long time with high rates of organic amendments the dispersion of large aggregates may be increased^13,16^, in the case of our study the decreased share of the >0.5 mm macroaggregate fraction in the soil of RotB might result mainly from the lower contents of bivalent cations, Ca^2+^ and Mg^2+^ (Fig. 4) in this soil compared to the soil in RotA, as these ions, particularly Ca^2+^, are known to play an important role in soil aggregation through the formation of bridges between clays and SOM particles^10,33^. Similarly to the results of previous studies^13,34^, FYM applied to the soil at the rate 40 Mg ha^−1^ increased the content of the >0.5 mm macroaggregate fraction and reduced the microaggregate fraction in comparison to the unmanured soil, but this effect was significant only in Rot A (Table 4).

**Table 4:** Contents of different aggregate size classes in air dry soil as influenced by crop rotation (I), manure application (II) and mineral N fertilizer (III) in the long-term field experiment at Grabow, Poland.

Crop rotation	Amendments		Aggregate content (g kg soil^−1^)		
	Manure (Mg ha^−1^)	N rates (kg ha^−1^)	>0.5 mm	0.5-0.25 mm	<0.25 mm
A (grain maize – winter wheat – spring barley – silage maize)	0	0	615.7	181.7	202.6
	0	100	617.7	172.0	210.3
	0	150	615.7	188.0	196.3
	Mean		616.3	180.6	203.1
	40	0	718.3	126.3	155.4
	40	100	700.3	146.0	153.7
	40	150	650.0	167.0	183.0
	Mean		689.6	146.4	164.0
	Overall mean		652.9	163.5	183.6
B (grain maize – winter wheat^†^ – spring barley – GCL^‡^)	0	0	517.0	281.0	202.0
	0	100	510.0	271.0	219.0
	0	150	526.3	249.0	224.7
	Mean		517.8	267.0	215.2
	40	0	539.3	272.0	188.7
	40	100	548.3	252.0	199.7
	40	150	508.3	277.7	214.0
	Mean		532.0	267.2	200.8
	Overall mean		524.9	267.1	208.0
LSD (*P* ≤ 0.05)			For: I = 30.4, II = 44.0, III = n.s. Interactions = n.s.	For: I = 12.5, II = n.s., III = n.s. Interactions = n.s	For: I = 16.0, II = 19.6, III = n.s. Interactions = n.s.

^†^Plus mustard green manure; ^‡^grass-clover ley.

In summary, this work has shown that the soil in RotB with an increased input organic matter (GM, GCL) accumulated significantly larger amounts of SOC and SMBC, had higher activities of dehydrogenase and acid phosphatase enzymes and gave significantly higher winter wheat grain yields compared to the soil in RotA without GM and GCL. However, the soil in RotB, contrary to that in RotA, has become more acidic, had lower contents of Ca and Mg and contained a diminished proportion of the >0.5 mm aggregates fraction. Thus, these results are in accordance with those reported earlier that arable crop rotations providing soil with larger amounts of organic matter, e.g. through growing GM or GCL, exert many beneficial effects on soil properties and productivity. Nevertheless, under some circumstances, e.g. in the absence of soil liming, such rotations may have also deteriorative effects on some properties (e.g. soil pH and aggregation) of loamy sandy soils (Luvisols). Regardless of the crop rotation, FYM applied once per 4-year rotation at the rate of 40 Mg ha^−1^ exerted beneficial effects on all the tested soil properties, including mitigating effects on the negative changes found in the soil of RotB.

## Methods

### Study site and experimental design

The study was based on a three-factor long-term field experiment, which has been established in 1980 at the Grabow Experimental Station (Lat: 51°21′N; Long: 21°40′E), belonging to the Institute of Soil Science & Plant Cultivation in Pulawy, Poland. The soil was classified as an Albic Luvisol (FAO, 1998) and is of loamy sand texture (70% sand, 25% silt, 5% clay). The climate at the site is temperate with a mean annual rainfall of about 560 mm and a mean annual temperature of 7.8 °C. The experiment includes two 4-years crop rotations (RotA and RotB) with the following order of crops: grain maize, winter wheat, spring barley and silage maize – for RotA and grain maize, winter wheat plus mustard GM, spring barley (with undersown grass–clover) and grass–clover ley (GCL) – for RotB. Till 2008 potatoes were grown in both rotations, which were then replaced by grain maize in view of the rapid expansion of grain maize in Poland. Barley straw and wheat straw are harvested in both rotations. Mustard (*Sinapis alba* L.) for green manuring is sown in the third decade of August, shortly after disk harrowing of winter wheat stubble. At the beginning of November green mustard biomass is disked and about two weeks later incorporated into the soil by ploughing. The grass–clover sward is harvested in three to four cuts per year and removed. Grass-clover sod is disked and ploughed down in the autumn. Within each rotation field, application rates of FYM and inorganic N fertilizer were varied in a split-plot design replicated in four blocks per field. Five FYM rates were assigned to main plots, starting in autumn 1979. FYM was applied in both rotations once per 4-year cycle, in the autumn preceding potatoes (grain maize), at rates of 0, 20, 40, 60 and 80 Mg ha^−1^. FYM was incorporated by disc harrowing and ploughing, one or 2 days after application. On average, FYM had a pH of about 8.4 and contained 5.4 (N), 3.5 (P_2_O_5_) and 5.8 (K_2_O) kg Mg^−1^ (fresh wt. basis). In 1988 N fertilizer rates were introduced as a third orthogonal factor. Four N rates were assigned to plots within each main plot, that is per FYM rate. Each plot measures 8 × 5 m gross, or 6.25 × 4 m net harvested area. In this experiment no soil liming has been done to show a “liming effect” of different rates of manure. Mineral fertilizes rates for particular plants are given in Table 1. N was applied as ammonium nitrate (34% N), P as triple superphosphate (45% P_2_O_5_) and K as potassium chloride (60% K_2_O). In this experiment conventional soil tillage system is used, with skimming in order to cover stubble, followed by mouldboard ploughing to 25 cm.

### Soil sampling and analyses

In 2013, when winter wheat was grown in RotA and RotB, replicated wheat plots from selected treatments (Tables 3 and 4) were sampled for the purpose of this study. At the beginning of June soil samples (ten per plot) were taken between plant rows from a 0–25 cm depth using a soil corer (30 mm internal diameter). Field moist soil samples were passed through a sieve with 2 mm openings and stored at 4 °C. Microbial biomass C was determined by the chloroform-fumigation-extraction method and calculated according to the following formula: C_mic_ = E_C_/k_EC_, where E_C_ = soluble C in fumigated samples – soluble C in control (un-fumigated) samples and k_EC_ = 0.45^35^. An Automated N/C Analyzer (Multi N/C 2100, Analytik Jena, Jena, Germany) was used to measure C contents in soil extracts. Dehydrogenase activity was estimated using TTC (2,3,5-triphenyltetrazolium chloride) as the substrate^36^ and, in the case of phosphatases (acid and alkaline), p-nitrophenyl phosphate (PNP) was used as the substrate^30^.

Subsamples of field moist soil were also sieved through 5 mm screens and air-dried at room temperature. These samples were used to assess basic parameters of soil structure (aggregation) using a dry soil sieving method similar to that described by Nimmo & Perkins^37^. To separate macroaggregates and microaggregates, portions (200 g) of air dried soil samples were hand sieved through a set of two nested sieves with 0.5 mm and 0.25 mm openings to obtain two macroaggregate size fractions (>0.5 mm and 0.5–0.25 mm) and one microaggregate fraction (<0.25 mm) collected in the bottom container. The aggregates retained in each compartment were weighed and expressed in g per kg of soil dry matter (DM).

Determinations of: C org. (PN-ISO 14235 – Soil Quality – Determination of organic carbon by sulfochromic oxidation), exchangeable Ca^2+^ and Mg^2+^ (extracted with 1.0 M ammonium acetate and measured by AAS) and soil pH (potentiometrically in 1:2.5 suspension of soil in H_2_O [ISO 10390 – Soil Quality – Determination of pH]) were performed by the certified chemical laboratory of the Institute of Soil Science and Plant Cultivation in Pulawy, Poland.

The data were subjected to the 3 way analysis of variance (ANOVA) with significance of differences assessed at *P* ≤ 0.05, using the FR-ANALWAR software based on Microsoft Excel.
